# A feasible probability and graph model for memory engrams

**DOI:** 10.3389/fncom.2026.1822722

**Published:** 2026-08-03

**Authors:** Hui Wei, Surun Yang, Yangwang Li

**Affiliations:** Laboratory of Algorithms for Cognitive Models, School of Computer Science, Fudan University, Shanghai, China

**Keywords:** capacity, directed graph, engram theory, long-term memory, probability model

## Abstract

The capacity of long-term memory seems to be extremely large, capable of storing information spanning almost a lifetime. Why does it have such a vast capacity? Why are some memories so enduring? What is the actual physical form of long-term memory? In the movie Inside Out, it is depicted as individual orbs containing information. Is that really the case? Simply explaining this by saying that the cortex has many neurons, numerous neural connections, and complex electrochemical activity between them is not sufficient to answer these fundamental questions. We need to uncover the theory hidden behind these phenomena. In essence, a neural network is equivalent to a very large directed graph, with a massive number of nodes and directed connections. This paper posits that the physical form of long-term memory is a connected subgraph within this complex directed graph. This subgraph is capable of linking together the disparate fragments of the same event, spread across different sensory cortices, to form associations. This provides a physical realization of the engram theory. The robustness of the connected subgraph and the resources it consumes can explain various memory behaviors. Based on anatomical, brain imaging, and electrophysiological evidence, this paper constructs a probabilistic connectivity model and uses theorems from graph theory to prove the ease of constructing connected subgraphs. Finally, it explains why the potential capacity for memory is immense.

## Significance statement

Understanding the physical basis of long-term memory remains one of neuroscience's deepest mysteries. This study proposes a novel theoretical framework that models the brain as a large directed graph, where each memory corresponds to a connected subgraph linking distributed sensory representations. By combining graph theory with neuroanatomical and electrophysiological evidence, the work provides a quantitative explanation for why long-term memory exhibits immense capacity and durability. This approach bridges abstract neural dynamics and concrete physical structure, offering a fresh perspective on how the brain organizes, stores, and stabilizes experience across time.

## 1 Introduction

Although the amount of information stored in a 1 TB hard drive is completely clear, the amount of information stored in a piece of cortex is quite obscure. Physical representations of digital formats, such as photos or text, prove to be hard drives or the floating gate of a solid-state drive, which are, so far, understood and delicately designed for information carriage. Quantifying storage for the brain remains unclear. If we dive deep into questions like “What is the storage format of a piece of information in the brain?”, “How much resource does storing a single piece of information consume?”, “How are these resources allocated?” and the like. When referring to mechanical formats, every single process of how they supplement information transformation is comprehended, key details for the process of the physical implementation form of semantic memory or episodic memory remain misty.

Storage, consolidation, and retrieval being its core functions, the memory system provides an information processing system that retains information, thereby altering future action modes. Storage refers to the formation of memories, ensuring future effective retrieval through encoding. Consolidation strengthens the durability of memories, assuring persistence for delayed retrieval. Retrieval involves reactivating stored information through clues, thus enabling recall or recognition. Speculations about the framework of the memory system have been made based on behavioral tests, to establish the multi-store memory model proposed by Atkinson (1968).

Episodic memory, being a representative long-term memory, involves various sensory channels as its components, such as visual, auditory, tactile, olfactory, gustatory, and spatial senses, to store event-related information. When a specific cue is activated, the entire context regarding the event is recalled. In research on context-dependent memory, Baddeley and Godden differentiated the contexts between the learning and recall phases in their tests. Experiment results indicated that participants performed better during the recall phase when they were positioned in a context similar to that of the learning phase (Godden and Baddeley, 1975). This suggested that a context similar to the learning phase promotes information retrieval and that the multiple aspects of an event are conjunctly encoded. How is this mechanism implemented? The Contextual Binding Model accounts for an explanation, which hypothesizes that when a memory is first processed by the hippocampus, the areas responsible for recognizing “what” the object is and “where” it is are connected to the hippocampus (Yonelinas et al., 2019). However, this explanation is still too vague to consider the details of how memory is physically represented.

In addition to behavioral science, neuroscience has also conducted meticulous research on memory, the engram theory being one of the current hotspots. The term “engram” was first proposed by Richard Semon in 1911, originally referring to changes in the brain induced by external stimuli, which lead to long-lasting effects (Semon, 1925). In modern neuroscience experiments, the engram transformation process is considerably represented at the somatic level. Classic studies relating engram cells suggest that initial engram forms in the hippocampus and gradually transport to the mPFC (medial Prefrontal Cortex) during consolidation. When long-term memory engrams are concreted, during the reactivation phase, neuronal signals travel through the mPFC-BLA (basolateral amygdala) pathway to elicit corresponding somatic receptors. For instance, in contextual fear conditioning, the shock response of mice to a conditioned stimulus proves identical with BLA engram activity (Josselyn and Tonegawa, 2020; Kitamura et al., 2017; Tonegawa et al., 2018). Experiments analyzing the concept of engram as entire ensembles have been studied, discovering that when a specific engram pathway is activated, various subregional ensembles are activated, and similar activations occur during slow wave sleep (Roy et al., 2022; Wang et al., 2023). It is suggested that the presence of a specific engram is not only physically represented by a particular ensemble in the hippocampus or BLA, but rather spreads to different parts of the entire brain. The hippocampus, BLA, or mPFC ensembles may serve as hubs that trigger the activation of the whole engram map, allowing one to recall stored details (Roy et al., 2022; Wang et al., 2023). When compared to the connections between non-engram cells, the synapses between engram cells exhibit morphological and functional specificity, which may prove morphological representation of memory retrievability (Choi et al., 2021; Hayashi-Takagi et al., 2015; Lee et al., 2023a,b). This collective biological evidence urges a plausible, indicative theoretical framework.

A powerful analytical toolchain provided by Marr's theory is suited to view memory as such an information processing system (Marr, 1982). An information system is suggested to be clarified with three levels of illustration: computation, algorithm, and implementation. The conclusions of behavioral experiments align with the computational level, demonstrating the functional features of memory. Neurobiological methodologies like MRI, EEG, somatic, or molecular analysis may serve as physical implementation. The algorithm remains unclear, serving as the missing link between the macro and micro levels.

At the behavioral level, studying the mechanisms of memory implementation can be too broad, while at the synaptic and molecular levels, it may be overly detailed. By abstracting neural networks into a massive directed graph, the encoding, consolidation, and retrieval of events can be viewed as the formation, strengthening, and activation of a connected subgraph within this directed graph. This approach acts as a bridge, linking together the complete chain of memory implementation. Through probabilistic modeling of neuronal connections and mathematical discussions on the existence of connected circuits, it is possible to reveal the immense capacity of biological neural networks in terms of large connected subgraphs. This could explain phenomena such as the large capacity, persistence, and forgetfulness of memory.

Directed graphs in graph theory may serve as an intermediate-level apparatus to connect neuronal details and psychological outcomes. This parallel distributed network may be presumed as the algorithmic implementation, thus complex electrochemical reactions between neurons are interactive activities between the nodes of directed graphs. Engrams, hence, might be interpreted as a connected subgraph within a directed graph, which representatively specifies a unique event. Memory encoding appears to involve the formation of such a connected subgraph, strengthening of synapses during consolidation in accordance with the strengthening of directed edges, to lift subgraphical stability, and retrieval relating to the reactivation of subgraphs stored previously. This approach fundamentally differentiates from using graphs to study functional connectivities between cortical regions, in which nodes are regarded as cortical or subcortical regions, edges as event-related connectivities, and graph algorithms as relevance revealed.

In graph analysis of brain networks, five generative undirected graph models are frequently utilized: ER random graphs, WS small-world networks, scale-free networks, spatial models, and stochastic block models (Lynn and Bassett, 2019).

Spatial models site the actual spatial location of neurons and fail to reflect the dynamic interactions and information transference between different brain regions. In contrast, small-world networks, ER random graphs, scale-free networks, and stochastic block models exhibit significant structural differences compared to data gathered from clinical experiments. Considering in-degree and out-degree asymmetry, it is hypothesized that the connection probability between neurons decreases exponentially as distance increases (Horvát et al., 2016; Perin et al., 2011; Pillai et al., 2012). Meanwhile, Kramer compared undirected graph generation models with clinical experimental data on amygdala neurons, and found significant discrepancies between the two (Kramer, 2023).

To conclude, to develop a directed graph network generation method that aligns with neurobiological details, with directed subgraphs physically representing engrams, and analysis of reachability, discrimination, and coexistence of these connected subgraphs supplies a plausible estimate of memory capacity.

## 2 Results

### 2.1 Memory engrams are essentially connected subgraphs

In neuroscience, neurons that are activated and involved in the storage of a specific memory during the process of memory formation are referred to as Engram Cells. Representing an event often requires a large number of cells, such as those widely distributed across different sensory channels, and these dispersed cells are connected through certain connecting units. This is analogous to a connected subgraph in graph theory. If we abstract the neural network into a highly connected, decentralized, and active directed graph, different Engram Cells would represent the nodes in the graph. When these nodes are connected, they form a connected subgraph within the vast directed graph of the brain. This connected subgraph could be the physical realization of a memory engram. Multiple connected subgraphs can coexist within the network, share some of the same nodes, and may lose parts of their branches due to resource competition. This physical realization can comprehensively explain the neurobiological implementation of the encoding, consolidation, and retrieval processes. It can also account for the conclusions drawn from psychological memory experiments.

### 2.2 Probabilistic model of cortical neuron connections

The connectivity properties of directed graphs must be supported by neurobiological experimental evidence. Anatomically, there is an observation that short-range connections between neurons are more common than long-range connections. However, this is not precise enough to construct large-scale directed graphs that approximate real neural networks. Biological neural networks are not trivial, unconstrained directed graphs, and the connectivity properties of such graphs must accurately reflect the objective reality of neural networks, at least statistically. To equate a neural network to a very large directed graph, we first need to establish the connection rules between each node in the graph. If the formation of synaptic connections by neurons at a specific location in space is considered an independent event, and if two neurons have enough synapses within a sufficiently small neighborhood, then it is possible for these two neurons to form synaptic connections within that neighborhood (Tecuatl et al., 2021). Based on these anatomical and electrophysiological findings, we derived a probabilistic model of neuron connections that reflects biological reality and verified that the model parameters are consistent with anatomical evidence. The connection probability between two neurons is a probabilistic function related to the distance between them. Through derivation (for details, see the Supplementary material: Neuron Connection Probability Modeling), the number of synapses *N*(*d*) formed between two neurons at a distance *d* can be expressed as a function of several parameters: the distance d between the two neurons, the distance *r* from the synapse formation site to the soma, a constant *A* (representing the proportion of upstream and downstream synapses that are likely to connect within a very small neighborhood), the synaptic density ρ_0_ at the center of the ellipsoid, and the synaptic density decay rate λ. All parameters and symbols used in the equations are defined in Table 1. This yields the following Equation 1:


$$\begin{array}{l} N\left(d\right)=A\rho_{0}^{2}\sum e^{-\frac{\sqrt{2\left(r^{2}+{\left(d-r\right)}^{2}\right)}}{\lambda }}\Delta r\; \end{array}$$ (1)

The connection probability between two neurons is determined by the number of synapses between them. Research has shown that the Poisson distribution can be used to fit the relationship between the potential number of synapses and the connection probability between neurons (Kersen et al., 2022) . The probability that a pair of neurons forms x synaptic connections is given by:


$$\begin{array}{l} P\left(x=n\right)=\frac{{\left(N\left(d\right)\right)}^{n}}{n!}e^{-N\left(d\right)} \end{array}$$ (2)

In reality, multiple synaptic connections may exist between two neurons. For example, the upstream axon terminal can form connections with various parts of the downstream neuron, such as the soma, the distal or proximal dendrites, or the axon hillock. When constructing a directed graph model in this paper, these multiple synaptic connections are aggregated into a single edge. Therefore, the connection probability between two neurons is the probability that there is at least one synaptic connection between them, which is the probability of being non-zero:


$$\begin{array}{l} P\left(be\text{\_}connected\right)=P\left(x\neq 0\right)=1-P\left(x=0\right)\\ \;=1-\frac{{\left(N\left(d\right)\right)}^{0}}{0!}e^{-N\left(d\right)}\\ \;=1-e^{-N\left(d\right)}\\ \;=1-e^{-A\rho_{0}^{2}\sum e^{-\frac{\sqrt{2\left(r^{2}+{\left(\left(d-r\right)\right)}^{2}\right)}}{\lambda }}\Delta r\;} \end{array}$$ (3)

Let $k=A\rho_{0}^{2}$,


$$\begin{array}{l} P\left(be\text{\_}connected\right)=1-e^{-k\sum e^{-\frac{\sqrt{2\left(r^{2}+{\left(d-r\right)}^{2}\right)}}{\lambda }}\Delta r\;} \end{array}$$ (4)

**Figure 1:**
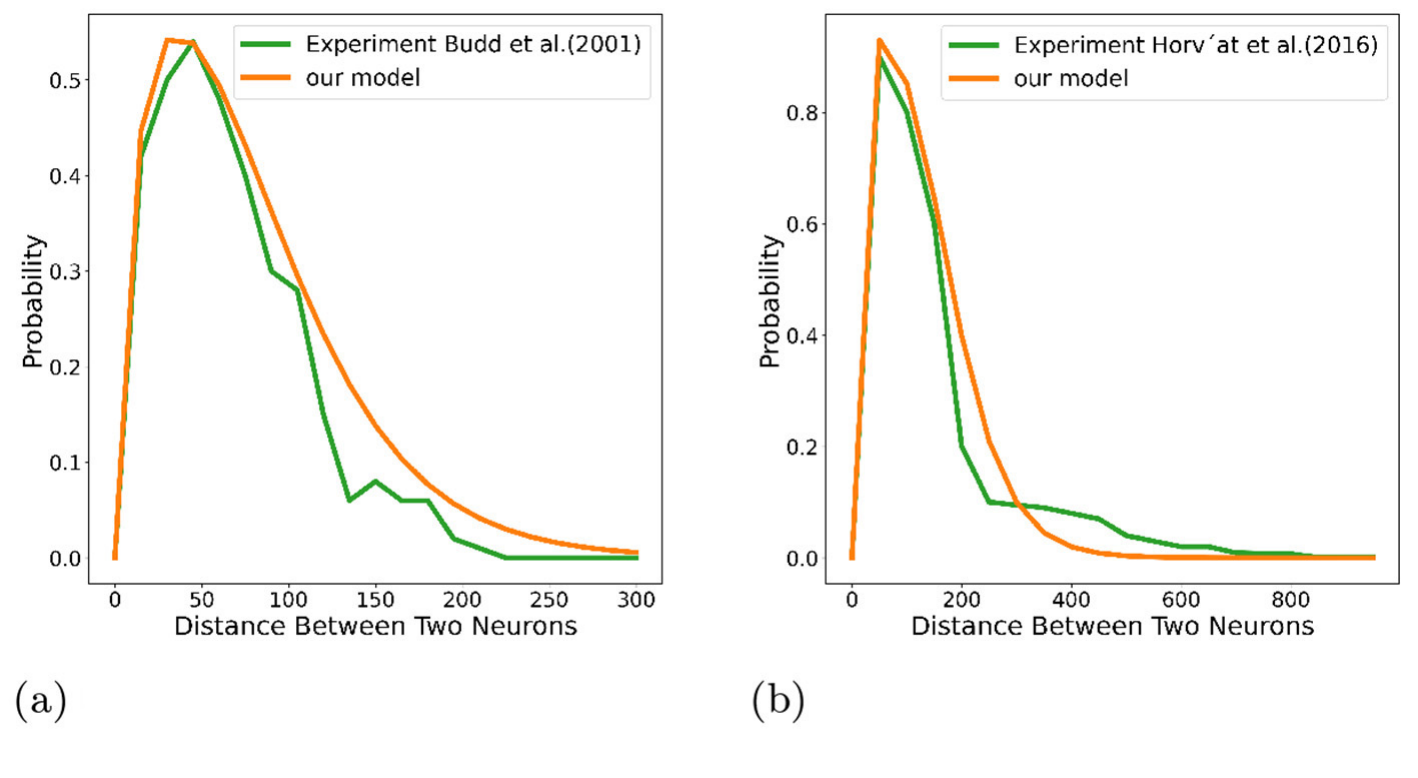
The function describing the connection probability between neurons, with the yellow curve representing our model and the green curve representing the experimentally measured data. **(a)** *k* = 0.06, λ = 41, compares the results from Budd. **(b)** *k* = 0.15, λ = 55, compares the results from Horvát.

Next, need to determine which set of parameters can generate a directed graph that aligns with biological reality. This paper selects a directed graph with 500 nodes for subsequent experiments (the reason for choosing 500 nodes can be found in the Supplementary material: Modeling Directed Graphs that Meet Biological Constraints). By adjusting different values of *k* and λ, various connection probabilities between cells observed in different biological experiments can be simulated. As shown in Figure 1a, the results are compared with the data from Budd and Kisvárday (2001), and in Figure 1b, the results are compared with the data from Horvát et al. (2016). These comparisons demonstrate the consistency between the theoretical parameters and actual experimental data. This consistency validates the parameters, which can then be used for further simulations of directed graph modeling.

**Table 1:** Model parameter.

Parameter	Meaning
*d*	The distance between the two neurons
*r*	The distance from the synapse formation site to the soma
ρ_0_	The synaptic density at the center of the ellipsoid
*A*	The proportion of upstream and downstream synapses that are likely to connect within a very small neighborhood
λ	The synaptic density decay rate
*N*(*d*)	The number of synapses formed between two neurons at a distance *d*
*P*(*X* = *n*)	The probability that a pair of neurons forms *X* synaptic connections

### 2.3 The mathematical guarantee of engrams is assured

A connected subgraph can serve as the physical implementation of an engram, provided that a directed graph has sufficient capacity, meaning it can accommodate a sufficient number of coexisting compatible connected subgraphs. Additionally, neurons activated by a particular event are dispersed throughout the cortex, and they should be connected with a relatively high success rate. For a set of nodes, if multiple connectivity schemes exist, then they can be easily connected. In graph theory, a Hamiltonian cycle refers to traversing all nodes in a graph and returning to the starting point. This theory can also be applied to a subset of nodes in a directed graph. Hefetz et al. (2016); Ferber et al. (2018) proved that for a random directed graph *D*(*N, p*), where *N* is the number of nodes and *p* is the independent probability of an edge existing (with at most *N*(*N*−1) edges), as long as *D*(*N, p*) satisfies the Asaf-Ferber condition: The in-degree and out-degree of any node are both non-zero, and *p*>log*N*/*N*, then:

Hamiltonian cycles must exist, and the number is typically *N*!(*p*(1+*o*(1)))^*N*^.

**Figure 2:**
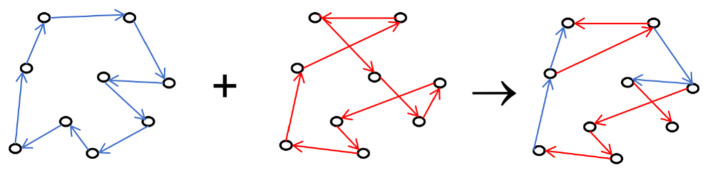
If a subset of nodes contains multiple Hamiltonian cycles, then connected subgraphs can be easily obtained.

A Hamiltonian cycle has stricter constraints than trivial connectivity, and this theorem characterizes the theoretical lower bound of graph connectivity, indicating the minimum that can be achieved. If a subset of nodes has more than one Hamiltonian cycle, it is very easy to ensure they are simply connected, as shown in Figure 2. If a directed graph's subgraph contains a sufficient number of Hamiltonian cycles, it indicates that activating a portion of the nodes in this subgraph can provide multiple stable pathways for the awakening and recall of the whole subgraph, which aligns very well with the initial motivation of engram theory.

So, do biological neural networks satisfy the constraints of this theorem? This paper uses unidirectional connection saturation to equivalently represent the connection probability *p* between nodes in a random directed graph. Unidirectional connection saturation is defined as shown in Equation 5, where *M* is the total number of edges in the directed graph, and *N* is the number of nodes in the directed graph. Unidirectional connection saturation can macroscopically characterize the connectivity of the entire set of nodes. For a set of nodes, if their unidirectional connection saturation is high, then these nodes have better connectivity, and the likelihood of a connection path existing between any two nodes is greater.


$$\begin{array}{l} {\left(Saturate\right)}_{unidirectional\;connection}=\frac{M}{N\left(N-1\right)} \end{array}$$ (5)

If the directed graph obtained according to Equation 4 (with parameters *k*=0.06 and λ=41) meets the Asaf-Ferber condition, the detailed experimental steps can be found in the section “Supplementary material: Experiment on the Relationship Between the Average Unidirectional Connection Saturation of Directed Graph Subgraphs and Subgraph Size”. The conclusions are as follows.

**Figure 3:**
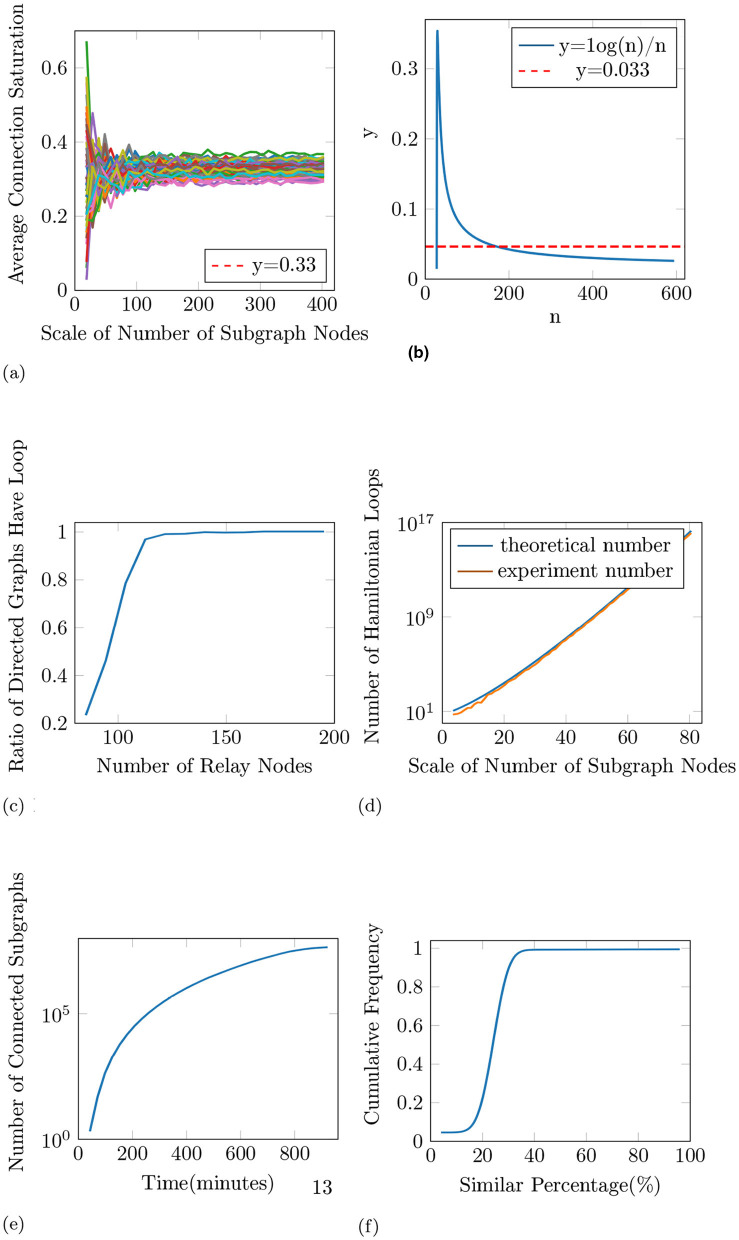
**(a)** The unidirectional connection saturation of directed graphs of different scales gradually stabilizes. **(b)** Comparison between *y* = log(*n*)/*n* and the empirical saturation level. **(c)** Ratio of directed graphs with different numbers of relay nodes forming cycles within two hops. **(d)** Theoretical and experimental values of the number of Hamiltonian cycles in the node set. **(e)** Sampling experiment verification shows a huge number of available connected subgraphs. **(f)** Subgraph similar percentage cumulative distribution function.

The statistical results of 80,000 directed graphs of different scales generated based on Equation 4 are shown in Figure 3a, with the x-axis representing the scale of the node set and the y-axis representing the average unidirectional connection saturation. From the figure, it can be observed that when the number of nodes exceeds approximately 50, the average unidirectional connection saturation of the node sets of different scales stabilizes around 0.033. Under the condition that parameters k and λ remain unchanged, it approaches a constant. Figure 3b shows the graph of *y* = log*N*/*N*. If we let log*N*/*N* < 0.033, then *N*>152.29. When *N* exceeds 1, log*N*/*N* continuously decreases as *N* increases, while 0.033 is a constant value. Therefore, theoretically, when the node set has 153 or more nodes, the directed graph will satisfy the Asaf-Ferber condition.

According to Equation 5, it is also possible to calculate how many Hamiltonian cycles exist when the node set has 153 or more nodes. Since finding Hamiltonian cycles in directed graphs is an NP-complete problem, the time complexity of traversal algorithms is very high, approximately *o*(*N*!), making it infeasible to obtain results in a reasonable time for larger *N*. To address this issue, this paper uses a computer to enumerate Hamiltonian cycles in directed graphs when the number of nodes is small. For larger node counts, the Monte Carlo method is used to estimate the number of Hamiltonian cycles present in the directed graph. The specific experimental strategy can be found in the section “Supplementary material: Counting Hamiltonian Cycles in Directed Graphs”.

The overall results are shown in Figure 3d where the theoretical values closely match the measured values. This experiment reveals that the magnitude of Hamiltonian cycles contained in the directed graph subgraphs generated by the biological neuron connection probability model, as well as the trend of variation in the number of cycles contained in subgraphs with different numbers of nodes, is similar to that predicted by the Asaf-Ferber formula. Thus, the directed graph model proposed in this paper theoretically possesses a substantial number of Hamiltonian cycles in its subgraphs, providing significant convenience for constructing connected subgraphs.

### 2.4 The formation cost of connected subgraphs is relatively low

In the subgraph of a directed graph, cycles can enhance the robustness of connections, forming closed-loop structures that ensure all relevant nodes are interrelated, preserving the possibility of being awakened and awakening others. The previous discussion has demonstrated that the subgraphs of the directed graph proposed in this paper, which meet biological constraints, theoretically possess a substantial number of Hamiltonian cycles. However, the detailed structural organization of different individuals' brains is not identical, particularly at the lower levels, such as the physical morphology of the neural circuit, yet they can still retain the same memories. This necessitates that directed graphs with different structural details can connect semantically similar nodes (such as neurons representing colors in the visual cortex or neurons representing audio in the auditory cortex) through different physical pathways.

At the same time, connectivity incurs costs, including hardware costs related to length and signal transmission time costs, which means that the connecting paths should not be excessively long. This can be represented using the concept of “hops” in graph theory. This paper experimentally verifies (see Supplementary material) the likelihood of initially activated nodes forming cycles within two hops, with the experimental results shown in Figure 3c. With relay nodes defined as intermediate nodes that are not necessarily part of the core node set of an event, but can participate in short directed paths that help connect otherwise weakly linked nodes into a connected subgraph, It can be observed that, under the current node connection probability, as long as there is a sufficient number of relay node, those initially activated nodes can be rapidly connected through local edges, thereby forming robust connected subgraphs. A more rigorous mathematical conclusion is that, under the premise of the connection probability defined in Equation 4, given *M* uniformly distributed nodes, the mathematical expectation of the distance scale between nearest neighbor nodes can be approximated as $\sqrt{s}$.Therefore, the probability that this subset of nodes fails to connect and forms two independent branches (where *M* = *M*_1_+*M*_2_) is:


$$\begin{array}{l} \overset{M_{1}}{\underset{x=1}{\prod }}{\left(1-P{\left(be\text{\_}connected\right)}_{d_{x}=J\sqrt{s}}\right)}^{M_{2}},J\in \left\{1,2,3,...,M\right\} \end{array}$$ (6)

This is a product of exponential functions, and when *M*≥20, the probability becomes very low. Furthermore, if the connection failure results in the formation of three isolated branches (where *M* = *M*_1_+*M*_2_+*M*_3_), the probability is:


$$\begin{array}{l} \;\overset{M_{1}}{\underset{x=1}{\prod }}{\left(1-P{\left(be\text{\_}connected\right)}_{d_{x}=J_{1}\sqrt{s}}\right)}^{M_{2}}\\ \times \overset{M_{1}}{\underset{x=1}{\prod }}{\left(1-P{\left(be\text{\_}connected\right)}_{d_{x}=J_{2}\sqrt{s}}\right)}^{M_{3}}\\ \times \overset{M_{2}}{\underset{x=1}{\prod }}{\left(1-P{\left(be\text{\_}connected\right)}_{d_{x}=J_{3}\sqrt{s}}\right)}^{M_{3}},\\ \;J_{1},J_{2},J_{3}\in \left\{1,2,3,...,M\right\} \end{array}$$ (7)

This value is also very small (see Supplementary material for details). Similarly, the probability of forming more isolated branches can be derived. Therefore, the probability of successfully connecting a set of nodes with a certain size is guaranteed.

### 2.5 The potential available capacity is enormous

A directed graph with a substantial number of nodes, when considering the combinations of nodes, will have an extremely large number of subgraphs. Based on the aforementioned connection probability between nodes, the likelihood of these subgraphs being connected is high. This implies that a directed graph can have an immense theoretical capacity. For example, in a directed graph formed by 540 nodes, if 20% of the nodes are selected to form connected subgraphs, the possible number of combinations is $C_{540}^{108}\approx 1.576\times 10^{85}$. If an event represented by 108 nodes is described by a statement composed of six words, the theoretical capacity limit would be equivalent to 1.17 × 10^77^ volumes of the Encyclopedia Britannica (digitized with text and images, approximately 6*GB*, assuming the average length of an English word is eight letters).

Furthermore, based on the discussion in 2.3, it is theoretically known that when the node set contains 153 or more nodes, the directed graph satisfies the Asaf-Ferber condition, meaning it contains at least one Hamiltonian cycle. Although 153 nodes can theoretically produce approximately 2 × 10^269^ different cycles, even in a directed graph with a unidirectional connection saturation of 3%, according to Asaf-Ferber's formula, there are still about 2 × 10^36^ Hamiltonian cycles, which is still an enormous number. Hamiltonian circuits impose overly strict requirements on connectivity, but in fact, weakly connected graphs can also serve as memory carriers. It is impossible for us to explore such a vast space within a limited time, and sampling is the only feasible experimental approach. Specifically, we perform random sampling of the edges between these 153 nodes and determine whether they can form weak connectivity. In a continuous 960-minute sampling experiment, we accumulated over 10^7^ connected subgraphs, as shown in Figure 3e. Notably, the number of connected subgraphs continues to grow, and the slope does not show signs of decreasing, indicating that this directed graph as a whole possesses immense potential capacity.

Considering distinguishability and the sparse usage of the combinatorial space, two subgraphs should maintain sufficient differentiation. If subgraph *A* and subgraph *B* have edge sets *Edge*_*A*_ and *Edge*_*B*_, the similarity between the two subgraphs is defined by the overlap ratio of their edge sets:


$$\begin{array}{l} Similar\left(A,B\right)=\left|\frac{{\left(Edge\right)}_{A}\cap {\left(Edge\right)}_{B}}{{\left(Edge\right)}_{A}\cup {\left(Edge\right)}_{B}}\right| \end{array}$$ (8)

Based on the aforementioned directed graph, if the similarity between any two subgraphs is required to be no greater than 40%, the experimental results, as shown in Figure 3f, indicate that approximately 55% of the subgraphs can meet this requirement. Although this significantly reduces the theoretical capacity, it still provides a considerable amount of practical usable capacity.

### 2.6 Incremental achievability

A directed graph can be seen as a collection of many stacked connected subgraphs, each representing a specific event through a particular topological structure Hui (2002); Wei and Luan (2004), This is fundamentally different from current machine learning methods (like the 2024 Nobel Prize in Physics and Chemistry), which bundle a large number of highly homogeneous and uniform samples, perform integrated learning, and use weights to represent them, essentially functioning as function approximation. The former allows for easy separation of the original samples, while the latter is difficult to separate, and the former provides better interpretability. The content of memory is incrementally added, making directed graphs more suitable for this “record-as-it-comes” method. Learning dispersed across the internal nodes can consolidate the topological structure of individual connected subgraphs Wei et al. (2023, 2024); Wei and Li (2023).

## 3 Methods

### 3.1 Mathematical model of connection probability between cortical neurons

Based on the synaptic density field, the number of synapses between two neurons was derived, and further, the expression for the connection probability between two neurons was obtained, as shown in Equation 4 (for details, see Supplementary material: Neuron Connection Probability Modeling).

### 3.2 Modeling directed graphs that meet biological constraints

Using the neuron probability model proposed in this paper as the basis for generating directed graphs, connectivity performance was tested with metrics such as reachability, average path length, and clustering coefficient for directed graphs with different numbers of nodes. It was found that directed graphs with 500 nodes exhibited superior connectivity performance. Therefore, a directed graph with 500 nodes was selected for subsequent experiments (for details, see Supplementary material: Modeling Directed Graphs that Meet Biological Constraints).

### 3.3 Experiment on the relationship between the average unidirectional connection saturation of directed graph subgraphs and subgraph size

Setting the directed graph parameters to *k* = 0.06 and λ = 41, the experiment was designed to statistically analyze the average unidirectional connection saturation of node subsets of different scales within the directed graph. The experiment confirmed that when the size of the node subset reaches a certain scale, the average connection saturation stabilizes around a fixed value (for details, see Supplementary material: Experiment on the Relationship Between the Average Unidirectional Connection Saturation of Directed Graph Subgraphs and Subgraph Size).

### 3.4 Counting hamiltonian cycles in directed graphs

When the number of nodes is small, computer enumeration was used to count the number of Hamiltonian cycles in the directed graph. For larger numbers of nodes, the Monte Carlo method was used to estimate the number of Hamiltonian cycles. The experiment verified that both methods produced approximately consistent results, with the experimental results shown in Figure 3d (for details, see Supplementary material: Counting Hamiltonian Cycles in Directed Graphs).

## Data Availability

The original contributions presented in the study are included in the article/Supplementary material, further inquiries can be directed to the corresponding author.
