# Supplementary material for: A feasible probability and graph model for memory engrams
Source: Front Comput Neurosci. 2026 Aug 3;20:1822722. doi: 10.3389/fncom.2026.1822722 (PMC13478142; doi:10.3389/fncom.2026.1822722)
Supplement: Supplementary file 1 [file Data_Sheet_1.pdf]

Supplementary Materials

Neuron Connection Probability Modeling

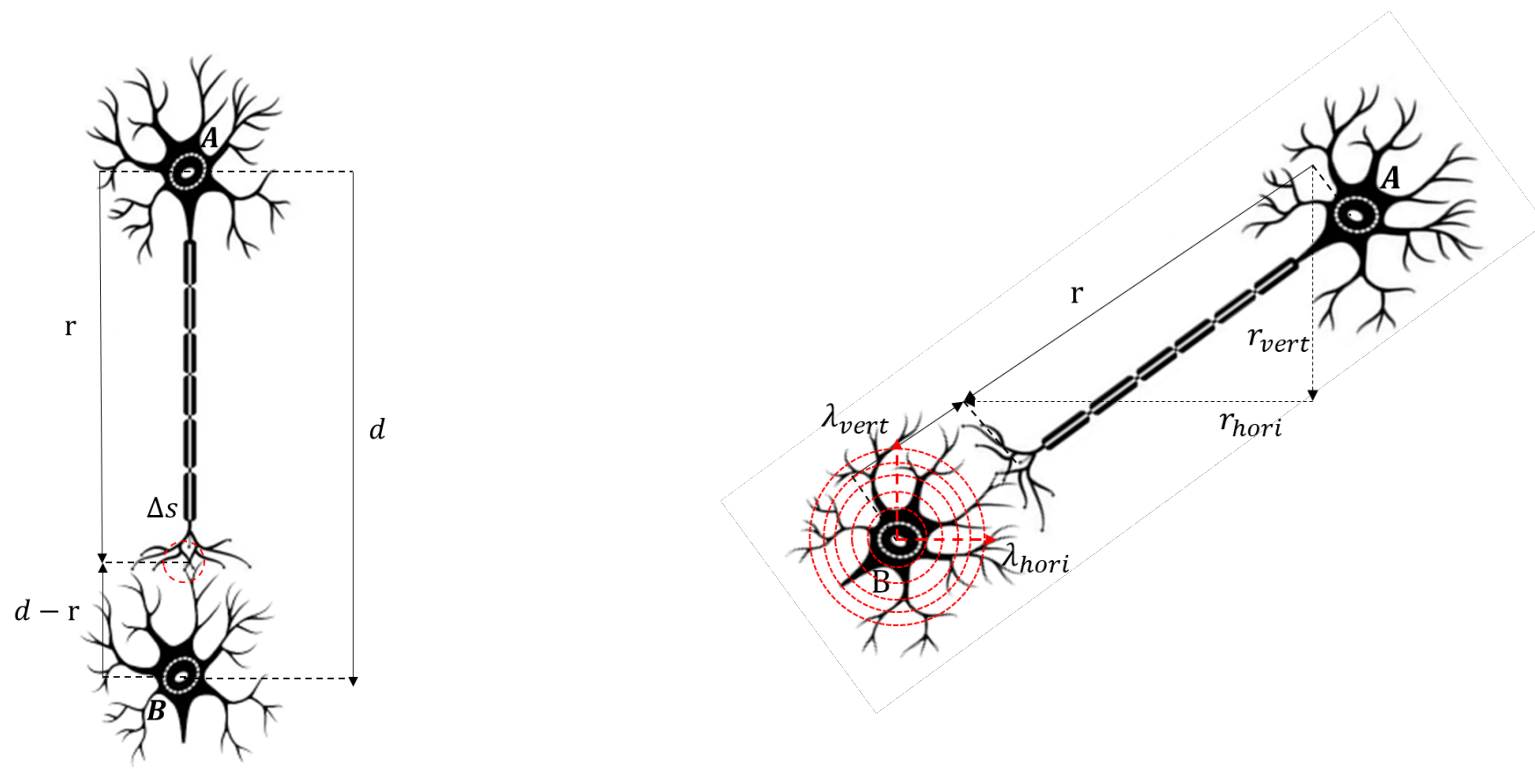

(a) Neighborhoods of Two Neurons (b) Diagram of Synaptic Field Density

Figure 4: Modeling of Neuronal Connections

Neurons connect to each other through synapses. If two neurons, A and B, have a sufficient number of synapses within a sufficiently small neighborhood, the probability of a connection between A and B increases. To determine the

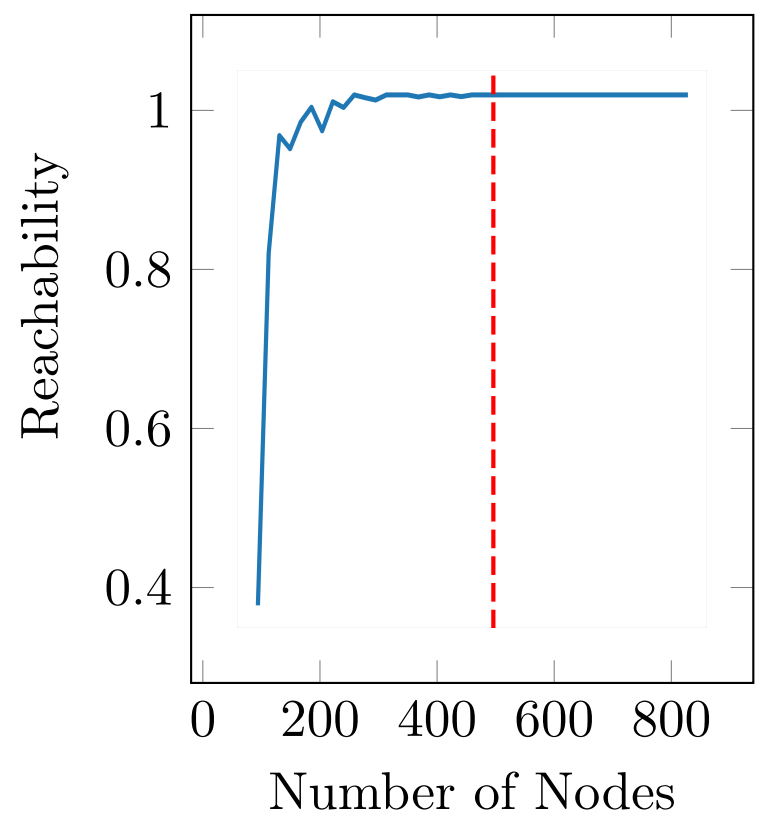

(a) Statistical results of reachability for directed graphs of different node sizes

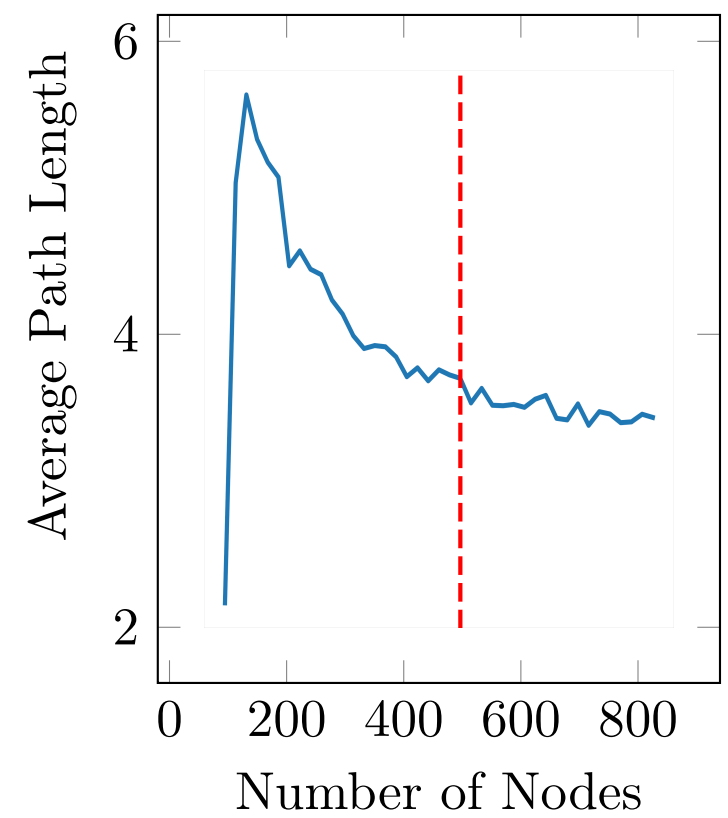

(b) Statistical results of average path length for directed graphs of different node sizes

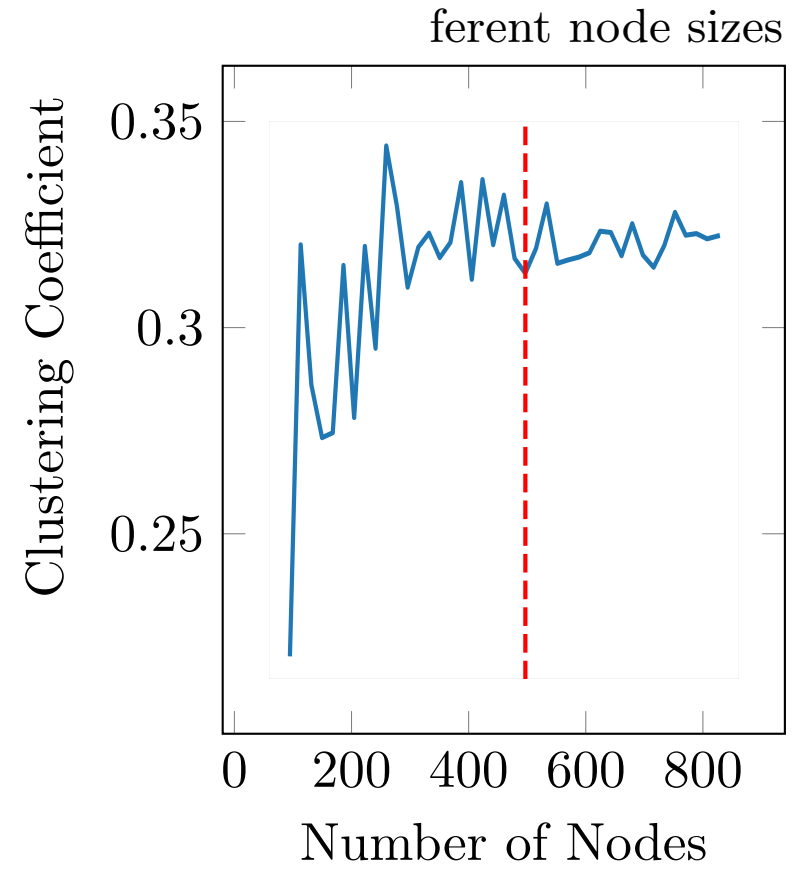

(c) Statistical results of clustering coefficient for directed graphs of different node sizes

Figure 5

probability of connection between any two neurons, whether they are near or far, this paper treats the formation of synapses at a particular spatial location as an independent event. if two neurons have enough synapses within a sufficiently small neighborhood, then it is possible for these two neurons to form synaptic connections within that neighborhoodTecuatl et al. (2021).

Since neurons are very small and can be approximated as thin layers with dendritic spines compressed into this layer, two-dimensional coordinates are sufficient to represent their positions. As shown in 4a, the distance between a presynaptic neuron A and a postsynaptic neuron B is denoted as d. Suppose neuron A is placed at the origin, and its axon has a length r. Neuron B is positioned at a distance d. They may have multiple neighborhoods close enough to form connections, and the location of each neighborhood depends on the values of d and r.

To represent the potential number of synapses in a certain neighborhood, the concept of synaptic field density is introduced Amirikian (2005). Synaptic field density refers to the density of synapses within a specific area, that is, the number of synapses per unit area. Let $\eta(r)$ be the number of synapses on the cell membrane of area Δs at distance r, and $\rho(r)$ be the synaptic field density. Then:

$$\rho(r) = \frac{\eta(r)}{\Delta s} \tag{9}$$

Let ξ be the proportion of upstream and downstream synapses that may connect to each other within the neighborhood Δs. If Δs is sufficiently small, then the number of synapses that upstream and downstream neurons can form at this location can be simplified to either 0 or 1 . Let $\eta(r)$ and $\eta(d-r)$ represent the number of synapses of neurons A and B in the neighborhood at r. Then, the density of the number of synaptic connections formed in Δs can be expressed

as:

$$\sigma(r,d) = \xi\ \frac{\eta(r)\cdot\eta(r-d)}{\Delta s} \tag{10}$$

Here, let $\Delta s \cdot\ \xi$ be considered as a constant A. Then, from 10., we have:

$$\begin{aligned}\sigma(r,d) &= \xi\cdot\Delta s\ \frac{\eta(r)\cdot\eta(r-d)}{\Delta s\cdot\Delta s}\\ &= A\frac{\eta(r)}{\Delta s}\ \frac{\eta(r-d)}{\Delta s}\\ &= \mathsf{A}\cdot\rho(r)\cdot\rho(d-r)\end{aligned} \tag{11}$$

The variable r represents the length covered by the axon and its terminals of the upstream neuron. The upstream neuron may have multiple axonal terminals that are in close proximity to the dendritic spines of multiple downstream neurons, thus forming connections within several small neighborhoods. Therefore, by integrating over r, we can obtain a function that represents the number of synaptic connections between upstream and downstream neurons:

$$N(d) = \mathsf{A}\int \rho(r)\cdot\rho(d-r)\,dr \tag{12}$$

Therefore, to further calculate $N(d)$, we also need to obtain the synaptic densities $\rho(r)$ and $\rho(d-r)$ at two locations. These can be approximated as follows: First, the cerebral cortex is a layered structure, and the physical properties of the cortex are isotropic in the horizontal dimension; thus, synaptic density can also be considered isotropic. Therefore, the synaptic density field can be modeled as an ellipsoidal field, as shown by the red dashed area in 4b. Second, research has found that the number of synapses decreases exponentially with respect to the longitudinal and transverse axes of the ellipsoid Sholl (1955). Thus, we can set

$$\rho(r) = (\rho)_0\, e^{-\sqrt{\frac{r_{hori}^2}{\lambda_{hori}^2}+\frac{r_{vert}^2}{\lambda_{vert}^2}}} \tag{13}$$

where ρ_0 is the synaptic density at the center of the ellipsoid, $\vec{r_{hori}}$ and $\vec{r_{vert}}$ are the horizontal and vertical components of $\vec{r}$, respectively, with $\vec{r} = \vec{r_{hori}} + \vec{r_{vert}}$. The decay rate is denoted by $\vec{\lambda}$, where $\vec{\lambda} = \vec{\lambda_{hori}} + \vec{\lambda_{vert}}$. To simplify the calculations, we can assume that the decay rates of synaptic density in the horizontal and vertical directions are the same, so $\lambda_{hori}^2 = \lambda_{vert}^2$, which gives $\left|\vec{\lambda_{hori}}\right| = \left|\vec{\lambda_{vert}}\right| = \sqrt{2}/2\vec{\lambda}$. Then,

$$\begin{aligned} \rho(r) &= (\rho)_0 \, e^{-\sqrt{\frac{\left(r_{hori}^2 + r_{vert}^2\right)}{\lambda_{vert}^2}}} \\ &= (\rho)_0 \, e^{-\sqrt{\frac{\left(r_{hori}^2 + r_{vert}^2\right)}{\frac{1}{2}\lambda^2}}} \\ &= (\rho)_0 \, e^{-\sqrt{\frac{2r^2}{\lambda^2}}} \end{aligned} \tag{14}$$

Thus, substituting into equation 12, have:

$$\begin{aligned} N(d) &= \mathsf{A} \int \rho(r) \cdot \rho(d-r) \, dr \\ &= \mathsf{A} \int \rho_0 e^{-\sqrt{\frac{(2r)^2}{\lambda^2}}} \cdot \rho_0 e^{-\sqrt{\frac{(2(d-r))^2}{\lambda^2}}} dr \\ &= \mathsf{A} \, (\rho_0)^2 \int e^{-\frac{\sqrt{2\left(r^2 + (d-r)^2\right)}}{\lambda}} dr \end{aligned} \tag{15}$$

Since equation 15 does not yield an analytical solution, we can only obtain a numerical solution for practical use, specifically by calculating the value of $N(d)$ for $r \in [0, d]$ This can be expressed as a definite integral as shown in equation16:

$$N(d) = \mathsf{A} \, (\rho_0)^2 \int_0^d e^{-\frac{\sqrt{2\left(r^2 + (d-r)^2\right)}}{\lambda}} dr \tag{16}$$

Using the first-order Euler approximation, the above definite integral can be transformed into the sum of the areas of multiple small rectangles, where Δr is

taken to be a very small value:

$$N\left(d\right) = \mathsf{A}\left(\rho_0\right)^2 \sum e^{-\frac{\sqrt{2\left(r^2+\left(d-r\right)^2\right)}}{\lambda}} \Delta r \tag{17}$$

Finally, we need to convert the possible number of synapses between two neurons into their connection probability. Research has shown that a Poisson distribution can be used to fit the relationship between the possible number of synapses and the connection probability between neurons Kersen et al. (2022), that is:

$$P\left(x = n\right) = \frac{\left(N\left(d\right)\right)^n}{n!} e^{-N(d)} \tag{18}$$

where x refers to the actual number of connections between neurons. Two neurons may have multiple synaptic connections, for example, the axon terminals of the upstream neuron may simultaneously form connections at various locations of the downstream neuron, such as the soma, the distal or proximal dendrites, or the axon hillock. In this paper, when modeling directed graphs, these synaptic connections are combined into a single edge, meaning that the connection probability between the two neurons is the probability that there is at least one synaptic connection between them, i.e., the probability is greater than zero:

$$\begin{aligned} P\left(be\_connected\right) &= P\left(x \neq 0\right) = 1 - P\left(x = 0\right) \\ &= 1 - \frac{\left(N\left(d\right)\right)^0}{0!} e^{-N(d)} \\ &= 1 - e^{-N(d)} \\ &= 1 - e^{-\mathsf{A}(\rho_0)^2 \sum e^{-\frac{\sqrt{2\left(r^2+(d-r)^2\right)}}{\lambda}} \Delta r} \end{aligned} \tag{19}$$

Let $k = A\left(\rho_0\right)^2$,

$$P\left(be\_connected\right) = 1 - e^{-k\sum e^{-\frac{\sqrt{2\left(r^2+(d-r)^2\right)}}{\lambda}}\Delta r} \tag{20}$$

Through the above derivation, the connection probability between neurons, influenced by factors such as the distance d between neurons, and the connection parameters k and λ, has been analytically characterized. Next, we can utilize this formula to further construct directed graphs that authentically reflect the findings of neurobiological experiments.

Modeling Directed Graphs that Meet Biological Constraints.

Using the neuron connection probability model proposed above as the basis for generating directed graphs, an enumeration experiment revealed that when the parameters are set to $k = 0.06$ and $\lambda = 41$, the neuron connection probability fits the data from Budd and Kisvárday (2001). Therefore, by setting the directed graph parameters to $k = 0.06$ and $\lambda = 41$, a directed graph model that meets biological constraints can be established. To test whether the generated directed graphs exhibit statistically significant connectivity, 400 directed graphs with different topological structures were generated within a 1000×1000spatial range, with node counts ranging from 100 to 900 in increments of 20. The connectivity performance was measured using three metrics: reachability, average path length, and clustering coefficient.

Reachability: A node B is considered reachable from node A if there exists a directed path that allows traversal from A to B. This paper assesses the reachability of the directed graph by calculating the percentage of nodes that can be reached on average.

Average Path Length: The average path length refers to the mean shortest path length between any two nodes in the graph. This paper evaluates the

average path length of the directed graph by computing the average of the shortest path lengths between all pairs of nodes.

Clustering Coefficient: For any given node v_i, the local clustering coefficient is defined as: $C(v_i) = e_i / k_i * (k_i - 1)$where e_i is the number of edges between the node and its neighbors, and k_i is the number of neighbors of the node. This paper uses the average local clustering coefficient of all nodes in the directed graph as an evaluation metric for the clustering degree of the graph.

Figure 5a shows the reachability statistics of directed graphs with different node scales. As the node distribution becomes denser, the reachability of the directed graphs also increases. Once the number of nodes reaches a scale of 500, the graph even becomes fully connected.

Figure 5b presents the average path length statistics of directed graphs with varying node scales. In scenarios with sparse node distribution, many nodes in the directed graph remain unconnected, resulting in a large number of nodes being unreachable. Only a few pairs of nearby nodes are connected, leading to a small average path length. As the scale of the nodes increases and the reachability of the directed graph improves, the average path length initially increases and then decreases, with the rate of decrease gradually slowing down, ultimately stabilizing around 3.6.

Figure 5c illustrates the clustering coefficient statistics of directed graphs with different node scales. When the node scale is small, the connectivity of the directed graph is poor, resulting in a low clustering coefficient. As the node scale increases, the clustering coefficient of the directed graph gradually stabilizes around 0.32. In the anatomical experiments conducted by Kramer et al. based on engram cells, results similar to theoretical predictions were observed Kramer (2023).

From the experiments evaluating the metrics of directed graphs with different

node sizes, it can be seen that a directed graph with 500 nodes exhibits high reachability, short average path length, and high clustering coefficient. This aligns with data from the cat visual cortex (area 17) in layer 4, where the average density of neurons is approximately 54,000 per cubic millimeter. Taking 1% of that, in a thin layer measuring 1000 μm $\times$ 1000 μm $\times$ 10 μm, there are about 540 cells. Since 10 micrometers is very thin and almost equivalent to a plane, it can be assumed that these 540 cells are distributed across this two-dimensional plane. The experiment also shows that a directed graph with 540 nodes possesses high reachability, short average path length, and high clustering coefficient, which facilitates information exchange between neurons. This density value represents an optimal choice. The theoretical analysis and anatomical evidence demonstrate good consistency, supporting the rationale behind the selection of parameters for the directed graph in this study. Therefore, a directed graph with approximately 540 nodes will be used for subsequent experiments.

The probability of a subset of nodes successfully forming a connected subgraph

Given the connection probability defined in 20, what is the probability that a subset of nodes (for example, a subset within the directed graph of 540 nodes mentioned above) successfully forms a connected subgraph? Assuming this subset has M nodes, intuition tells us that if M is too small, it indicates that the nodes are sparsely distributed and too far apart from each other. According to 20, the probability of them forming a connected subgraph would be low. Conversely, if M reaches a sufficient quantity and is uniformly distributed, the probability of them ultimately achieving connectivity would be significantly higher.

Assuming M nodes are uniformly distributed among the aforementioned 540 nodes, they can form a layout similar to a super-pixel partition, where the area

of each super-pixel is s=1000μm*1000$\mu m/M$. The two nearest neighbor nodes can be approximated to be at the endpoints of the longer diagonal of the super-pixel, so the mathematical expectation of the distance scale between them can be approximated as $\sqrt{s}$. With this neighboring distance value, the connection probability between the two nearest neighbor nodes is $P(be\_connected)_{d=\sqrt{s}}$.

The probability of M nodes being connected equals "1 - the probability of having two isolated branches - the probability of having three isolated branches - ... - the probability of all M nodes being isolated." For example, to form two isolated branches, let branch 1 contain M_1 nodes and branch 2 contain M_2 nodes, where $M = M_1+M_2$. The probability that any node in branch 1 does not connect to any node in branch 2 is: $\prod_{x=1}^{M_1}(1 - P(be\_connected)_{d_x=J\sqrt{s}})^{M_2}, J \in \{1, 2, 3, ..., M\}$. This is a product of exponential functions, and when $M \geq 20$, the probability becomes very low. Furthermore, if the connection failure results in the formation of three isolated branches (where $M = M_1 + M_2 + M_3$), the probability is:

This value is also very small (see supplementary information for details). Similarly, the probability of forming more isolated branches can be derived. Therefore, the probability of successfully connecting a set of nodes with a certain size is guaranteed.

$$\begin{aligned}
&\prod_{x=1}^{M_1}(1 - P(be_connected)_{d_x=J_1\sqrt{s}})^{M_2} \\
&\times \prod_{x=1}^{M_1}(1 - P(be_connected)_{d_x=J_2\sqrt{s}})^{M_3} \\
&\times \prod_{x=1}^{M_2}(1 - P(be_connected)_{d_x=J_3\sqrt{s}})^{M_3}, \\
&J_1, J_2, J_3 \in \{1, 2, 3, ..., M\}
\end{aligned} \tag{21}$$

Experiment on the Relationship Between the Average Unidirectional Connection Saturation of Directed Graph Subgraphs and Subgraph Size

In the experiment, the directed graph parameters are set to k=0.06 and λ=41. To ensure the statistical significance of the experimental results, the experimental scheme is as follows:

1. Generation of Directed Graphs: Generate 500 nodes uniformly distributed within a 1000×1000 area. Randomly create 100 directed graphs, all with the same parameters k and λ, but differing in the details of their topological structure.

2. Subgraph Sampling: For each directed graph, vary the number of nodes from 10 to 300 in increments of 10, generating 40 different scales of subsets. For each scale, randomly select 20 subset samples and compute the average unidirectional connection saturation for each group.

3. Observation and Analysis: After conducting experiments on the 100 directed graphs, observe the average unidirectional connection saturation of subgraphs at different scales of node subsets. The statistical results of 80,000 directed graphs of different scales are shown in Figure 3(a), with the x-axis representing the scale of the node set and the y-axis representing the average unidirectional connection saturation. From the figure, it can be observed that when the number of nodes exceeds approximately 50, the average unidirectional connection saturation of the node sets of different scales stabilizes around 0.033.

Counting Hamiltonian Cycles in Directed Graphs

Scheme 1: When the number of nodes $N \leq 20$, use a computer to traverse the directed graph and count the number of Hamiltonian circuits:

1. Randomly generate 1000 directed graphs with N uniformly distributed

nodes, ensuring that the unidirectional connection saturation of the directed graph is greater than $\log N/N$, with parameters k and λ being random.

2. Use the traversal method to calculate how many Hamiltonian circuits exist in one directed graph, then compute the average and compare it with $N!\left(Saturate_{unidirectional\ connection}\right)^N$.

Scheme 2: When the number of nodes $N \geq 20$, use the Monte Carlo method for estimation: 1. Randomly generate 1000 directed graphs with N uniformly distributed nodes, ensuring that the unidirectional connection saturation of the directed graph is greater than $\log N/N$, with parameters k and λ being random. 2. For each selected directed graph, if the number of nodes is N, randomly take M different permutations of these N nodes as samples. Then, sequentially check whether these permutations correspond to a traversal order of nodes that forms a Hamiltonian circuit in the directed graph. If a permutations match this condition, then the number of Hamiltonian circuits in that directed graph can be estimated as $a/M \times (N!)$. 3. Compare this result with $N!\left(Saturate_{unidirectional\ connection}\right)^N$. The experimental results obtained from the two schemes are shown in Figure 3(d), where the measured values approximate the trend calculated using Asaf-Ferber's formula.
